# Western influenced lifestyle and Kv2.1 association as predicted biomarkers for Tunisian colorectal cancer

**DOI:** 10.1186/s12885-020-07605-7

**Published:** 2020-11-10

**Authors:** Mouadh Barbirou, Henok G. Woldu, Ikram Sghaier, Sinda A. Bedoui, Amina Mokrani, Radhia Aami, Amel Mezlini, Besma Yacoubi-Loueslati, Peter J. Tonellato, Balkiss Bouhaouala-Zahar

**Affiliations:** 1 Laboratory of Venoms and Therapeutic Biomolecules, LR16IPT08 Institute Pasteur of Tunis, University of Tunis El Manar, 13 Place Pasteur, BP74, 1002 Tunis, Belvédère Tunisia; 2 Center for Biomedical Informatics, School of Medicine, University of Missouri, Columbia, MO USA; 3 Department of Health Management and Informatics, Biostatistics & Research Design Unit School of Medicine, University of Missouri-Columbia, Columbia, MO USA; 4 University of Tunis El Manar, Tunis, Tunisia; 5 Laboratory of Mycology Pathologies and Biomarkers Faculty of Sciences of Tunis, University of Tunis El Manar, Tunis, Tunisia; 6 Medical Oncology Division, Salah Azeiz Oncology Institute, University of Tunis El Manar, Tunis, Tunisia; 7 Medical School of Tunis, University of Tunis El Manar, Tunis, Tunisia

**Keywords:** Colorectal Cancer, Western lifestyle, *KCNB1*, Polymorphisms, Cancer prevention

## Abstract

**Background:**

Colorectal cancer (CRC) is the third most diagnosed malignancy worldwide. The global burden is expected to increase along with ongoing westernized behaviors and lifestyle. The etiology of CRC remains elusive and most likely combines environmental and genetic factors. The Kv2.1 potassium channel encoded by *KCNB1* plays a collection of roles in malignancy of cancer and may be a key factor of CRC susceptibility. Our study provides baseline association between Tunisian CRC and interactions between *KCNB1* variants and lifestyle factors.

**Methods:**

A case-control study involving 300 CRC patients, and 300 controls was conducted Patients were carefully phenotyped and followed till the end of study. *KCNB1* genotyping was confirmed by Sanger sequencing. Bivariate and multivariable logistic regression analyses were used to assess the clinical status, lifestyle and study polymorphisms association with CRC.

**Results:**

We noted significant gender association with CRC occurrence. Moreover, CRC risk increases with high meat and fat consumption, alcohol use and physical activity (PA). Carriage of rs1051296 A/G and both rs11468831 ins/del and del/del genotypes (*p* < 0.001) were significantly associated with CRC risk. Analysis according to gender reveals correlation of rs1051295 A/G, rs11468831 non ins/ins (*p* = 0.01) with CRC susceptibility regardless of patients’ gender while rs3331 T/C (*p* = 0.012) was associated with females. Stratification study according to malignancy site; Rectal Cancer (RC) and Colon Cancer (CC), reveals increasing RC risk by gender and high meat and fat consumption, alcohol use and PA. However, additional association with high brine consumption was noted for CC. The rs1051295 A/G (*p* = 0.01) was associated with RC risk. Increased CC risk was associated with carriage of rs1051295 A/G, rs11168831 (del/del) and (ins/del) genotypes.

**Conclusion:**

The risk of CRC increases with modifiable factors by Western influences on Tunisian lifestyle such as alcohol use, high fat consumption and possibly inadequate intake of vegetables. In addition, *KCNB1* polymorphisms also markedly influence CRC susceptibility. Our study establishes key elements of a baseline characterization of clinical state, Western influenced lifestyle and *KCNB1* variants associated with Tunisian CRC.

## Background

There has been a dramatic increase of colorectal cancer (CRC) incidence and mortality rates around the world. Now, CRC is the third most diagnosed cancer with 1.8 million new cases and the second leading cause of malignancy death [1]. Although CRC develops in the large intestine, it is characterized as a highly heterogeneous disease often arising without family history or significant evidence of mutations that often increase cancer risk [2]. In addition, CRC lifestyle risk factors (e.g. increased meat and fat consumption and alcohol and tobacco use) demonstrate further examination of proteins and genetics associated with key digestive tract function. Overall, CRC etiology likely includes both somatic genetic and epigenetic aberrations possibly confounded by such lifestyle factors [3, 4]. Though CRC is known as a malignant disease of developed countries, recent studies demonstrate that the healthcare and incident burden is now increasing in low and middle income countries during economic development and corresponding changes from traditional to lifestyle influenced by western culture [5].

In 2018, the prevalence of CRC in Tunisia was estimated at 6.3/100,000 [2]. A 2017 review of the North-Tunisia Cancer Registry (NTCR) demonstrated a significant increase in CRC incidences since 1994 and predicted a 2024 incidence rate of 39.3/100,000 for male and 22.9/100,000 for female in the absence of a robust screening program [6]. The unique geographical and historical position of Tunisia at the crossroads of the African, Asian, and European cultures and people provides an excellent platform to study the genetic background of a mosaic genetic population undergoing relatively recent western lifestyle influences across a collection of ethnicities.

In previous gene focused studies, several somatic mutations in known CRC driver genes have been identified (namely, *APC*, *KRAS*, *TP53* and *BRAF*) [2] and GWAS (Genome-Wide Association Studies) continue to elucidate CRC pathogenetic mechanisms by identification of associated genes and suggest collection of genes of common function possibly important in targeted mechanisms associated with the development of CRC. Recently ion channel genes expression was extensively examined in gastrointestinal cancer cells demonstrating modulation and/or regulation of normal and cancer stem cells [7, 8]. The potassium (K+) channels, present the most diverse class of ion channels, are involved in activation, apoptosis and several physiological functions as well as in the regulatory mechanisms of neoplastic cell proliferation and survival [9, 10]. It is well established that K+ channels exhibit oncogenic properties and have been linked to a more malignant cancer phenotypes [9, 10]. Of many K+ channels, the voltage-gated potassium channel family (Kv) plays important roles in differentiation and growth of excitable cells. Inhibition of Kv channels also leads to suppressed proliferation of various cancer cells [8]. Furthermore, studies have linked apoptosis to potassium ion loss via cytoplasmic K+ decrease thus enhancing caspase and nucleases activation that cause apoptosis [11–13]. Although significant evidence suggests a prominent role for Kv channel, the role of such channels has remained largely unexplored in CRC. One promising, functionally well characterized Kv candidate, Kv2.1 (coded by *KCNB1* gene) [14], has demonstrated a role in several cancers including gastric [15] and endometrial cancer [16]. Kv2.1 forms complexes with other homo-multimer functional channels such as Kv9.3 that modulate the electrophysiological properties of excitable cells and demonstrate a potential oncogenic role [16]. Consideration of such protein families’ role in apoptosis regulation suggests that potassium channels are regulators of cancer cell death and may lead to promising therapeutic targets. In particular, a recent study by this group provides evidence about the association of *KCNB1* polymorphisms (rs3331, rs1051295 and rs11468831) and treatment response and may help predict if not contribute to anticancer treatment response, a part of a new approach for monitoring the disease kinetics during the chemotherapy process [17].

This collective evidence suggests that potassium channels may play an important role in CRC risk, development and metastasis and that Kv2.1 in particular, through modified expression attributed to *KCNB1* mutations (rs3331, rs1051295 and rs11468831) [18] is a notable candidate of the potential role of potassium channel proteins in CRC.

The primary aim of this study is to test whether specific *KCNB1* polymorphisms influence the outcomes of CRC among Tunisian patients. The second objective of this study is to examine the possible links between CRC associated genetic variants and western influenced changes of Tunisian lifestyle (including meat, fat, vegetable and brine consumption and tobacco, alcohol and physical habits) observed during CRC increased occurrence in the Tunisian population.

## Methods

### Study subjects

A retrospective case-control study was conducted between January 2015 and December 2018, at the outpatient oncology service of Salah Azaiz Hospital in Tunisia. A total of 300 subjects with CRC and 300 cancer-free subjects were recruited into the study. None of the control subjects reported personal or family history of CRC, nor any other complex disease including arterial hypertension, hyperglycemia, and anemia. Healthy controls were matched according to self-declared ethnic origin, age and BMI.

Table 1 lists subjects’ characteristics. CRC subjects underwent complete clinical examination including colonoscopy and biopsy histopathology to clinically confirm diagnosis. A detailed questionnaire was administered to assess subject lifestyle including diet, drug, physical activity, tobacco and alcohol use. Clinical data was gathered from clinical records retrospectively and updated by routine medical checkup that included review and testing of anemia, hyperglycemia, hypertension, tumor pathological report (size, localization, stage, differentiation status and histological type), CA19–9/CAE measurement and *KRAS* status. Medical status of controls was confirmed by an annual medical examination, a face-to-face interview and review of the same lifestyle questionnaire as given to cases. All cases and controls digital records were anonymized and entered into a study data base (“DCP DB”).

**Table 1:** Participant’s characteristics (Cases and Controls)

Parameters	Cases ***N*** = 300 (%)	Controls ***N*** = 300 (%)	Total ***N*** = 600 (%)	P ^**1**^
**Demographic**				
**Gender** (*Male / Female*)	146/154	91/209	237/363	**< 0.001**
**Age** (*years*) ^a^	56.80 ± 10.22	55.30 ± 12.20	56.01 ± 11.23	0.09
**BMI** ^a^	25.04 ± 45.08	25,60 ± 5.14	25.51 ± 5.10	0.75
**Lifestyle**				
Vegetable consumption (high/low)	282/18	278/22	560/40	0.51
Brine consumption (high/low)	199/101	187/113	386/214	0.30
Meat consumption (high/low)	196/104	267/33	463/137	**< 0.001**
Fat consumption (high/low)	271/29	165/135	436/164	**< 0.001**
Tobacco use (never/ sometimes)	190/110	237/63	427/173	**< 0.001**
Alcohol use (never/ sometimes)	215/85	264/36	479/121	**< 0.001**
Physical activity level (high/low)	31/269	233/67	264/336	**< 0.001**
**Clinical History**				
Hypertension	223 (74.30)	NA	NA	NA
Hyperglycemia	74 (24.70)	NA	NA	
Anemia	73 (24.30)	NA	NA	
***Tumor localization***				
Colon	190 (63.30)	NA	NA	NA
Rectum	110 (36.70)	NA	NA	
***Histological type (Adenocarcinoma)***				
Lieberkuhnion	243 (81)	NA	NA	NA
Tubular	21 (7)	NA	NA	
Mucinous	32 (10.70)	NA	NA	
Signet Ring Cell	4 (1.30)	NA	NA	
***Differentiation***				
Poor	26 (8.70)	NA	NA	NA
Moderate	109 (36.30)	NA	NA	
Well	165 (55)	NA	NA	
***TNM classification***				
I	1 (0.30)	NA	NA	NA
II	123 (41)	NA	NA	
III	120 (40)	NA	NA	
IV	56 (18.70)	NA	NA	
***Treatment***				
Surgery	2 (0.70)	NA	NA	NA
Chemotherapy	38 (12.70)	NA	NA	
Surgery+ Chemotherapy	190 (63.30)	NA	NA	
Chemotherapy+ Radiotherapy	22 (7.30)	NA	NA	
Mixed	48 (16)	NA	NA	

*BMI* Body Mass Index, *TNM* Tumor, Nodes, Metastases according to Dukes Classification modified by Astler-Coller; Mixed: surgery + chemotherapy + radiotherapy, *NA* Not Applicable

^1^ Pearson chi square (categorical variables), Student t-test (continuous variables), Value in bold is statistically significant < 0.05

^a^ mean ± standard deviation

### Lifestyle data

Interviews of all cases (*N* = 300) and controls (*N* = 300) was performed by a trained study interviewer using a structured French language questionnaire including questions on demographics, individual and family general medical history, individual and family cancer history and lifestyle including daily and weekly diet, tobacco, alcohol and physical activity. Subject’s height and weight were measured before surgery for cases and during an annual health examination for controls. Average dietary intake was investigated by a semi-quantitative food frequency set of questions. Individuals were categorized as “high” meat consumers if total “meat” (the total of red and processed meat, fish and poultry) consumed was at least 150 g/day.

The “high” fat consumers were defined as those who consumed greater than 200 g of animal fat, oil, butter, cheese and dry fruit, 3 or more times a week. Individuals were classified as “high” fruit and vegetable consumers if the frequency was 5 or more times a week and were categorized as “high” brine (processed vegetable, fruit and meat) consumers if subject consumed at least 150 g/day 3 or more times a week. Tobacco use (including hookah and cigarette smoking) status (“never-used”, “former user”, or “current user”), age of first tobacco use, the period of smoking and number of cigarettes/hookahs per day was recorded. For the purpose of this study, “former user” and “current user” were categorized as tobacco “user”. Alcohol use status (“never-drinker”, “former drinker” or “current drinker”), and the average frequency of consumption was recorded. For the purpose of this study, “former drinker” and “current drinker” were categorized as alcohol “user”. Physical activity was assessed by number of hours per day and days per week. “high” physical activity level was defined by more than 2 hours per day and at least 5 days a week. The nature of the subject’s work (physical or non-physical) was taken into consideration when defining “high” physical activity level. All lifestyle and habit factors included in this study were categorized consistent with the principles defined in the PhenX Toolkit TRIG system [19]. All subjects’ questionnaire answers were transcribed and validated by trained project personnel and recorded in the DCP DB.

### Assessing the functional relevance of KCNB1 genetic variations

The potential functional implications on the Kv2.1 protein of two *KCNB1* gene variants and one related indel were predicted using SNPnexus [20]. FitCons and GWAVA non-coding variants functional predictions provided by SNPnexus were examined. The fitCons calculations give a probability of mutational “fitness consequences” which integrates information from both evolutionary data and functional genomic data (RNA-seq, ChIP-seq and DNase-seq) and is especially suited at quantifying the effect of mutations on cis-regulatory elements in noncoding DNA [21]. The fitCons score is considered significant at *p* < 0.003 (“highly significant score”) with higher scores indicating more likely genomic functional modification [22]. GWAVA produces three scores (Region, TSS and Unmatched) from three different approaches. As in previous studies [23], we use a threshold of any one of the three scores, > 0.5 to suggest variants with likely functional implications.

### KCNB1 genotyping

Genomic DNA was prepared by QIAamp® DNA blood Mini Kit, according to the instructions of the manufacturer (Qiagen GmbH, Hilden, Germany). Genotyping of two SNPs, located in *KCNB1*’s 3’UTR (rs3331, rs1051295), was performed using ARMS-PCR and verified by direct Sanger sequencing. While the nearby insertion deletion (rs11468831) was simple PCR amplified followed by capillary electrophoresis on the Agilent 2100 Bioanalyzer (Agilent 2100 Bio-analyzer, Biotech Vertriebsgesellschaft m.b.H Agilent Technologies, Lithuania). All genotypes, sequences and controls were validated by trained project personnel and recorded in the DCP DB.

### Statistical analysis

All data for the study was recorded and stored into the study database (DCP DB). Data quality and overall assessment was performed by a trained statistician expert in study data review. Typos, artifacts, missing data and abnormal statistics were assessed by the project team and decisions to correct data approved after original source review. Decisions to exclude data or subject records were conducted by the data analysis team consisting of the Study lead, operational director and statistical lead. After data review, ‘approved’ data and records were notated for subsequent statistical analysis. Distribution of the clinical characteristics of the CRC patients was summarized using frequency and percentage. Two sample T-test and Pearson Chi-square test were used to assess differences in the demographic and lifestyle characteristics between the CRC patients and Controls for continuous and categorical variables respectively. Bivariate and multivariable logistic regression models were fit to the CRC and separately to the RC and CC cohort data to assess whether demographic, lifestyle or *KCNB1* related genotypes are associated with CRC, RC or CC. Models were fit after adjustment to lifestyle factors to test the robustness of the associations. In addition, we tested association *KCNB1* haplotype against all factors. From each fitted model, odds ratios (ORs), corresponding 95% confidence intervals and adjusted *p*-values were extracted. All calculated p-values were two-sided and p-values less than the predefined significance level (alpha = 0.05) were considered statistically significant. All statistical analyses was performed using RStudio (Ver. 1.1.456) and R (Ver. 2.14.0) [24, 25].

## Results

### Clinical

The clinicopathological characteristics of CRC subjects (Table 1.) are including hypertension (74.30%), anemia (24.30%) and diabetes (24.70%). Most CRC cases present as Lieberkuhnian adenocarcinoma (81%), well differentiated (55%) and at the colon site (63.30%). CRC Dukes staging assigned according to TNM classification of the UICC was I (0.30%), II (41%), III (40%) and IV (18.70%). A majority of the CRC subjects were treated surgically followed by chemotherapy (63.30%). Controls had no incidences of cancer or clinicopathologies and as expected from the matched study design, mean age and body mass index (BMI) were not statistically different between the CRC cases and controls (*p* > 0.09) though gender frequencies were statistically different *p* < 0.001, (Table 2).

**Table 2:** Association of lifestyle and *KCNB1* related genotype with CRC, CC and RC risk

***Parameters***	Total population (***N*** = 600)				stratification according to tumor site							
	Colorectal cancer (N = 300)				Colon cancer (***N*** = 190)				Rectal cancer (***N*** = 110)			
	***Bivariate***		***Multivariable***		***Bivariate***		***Multivariable***		***Bivariate***		***Multivariable***	
	OR (95%CI)	P	OR (95%CI)	P^**a**^	OR (95%CI)	P	OR (95%CI)	P^**a**^	OR (95%CI)	P	OR (95%CI)	P^**a**^
Age (years)	1.01 (1.00–1.03)	0.098	–	–	1.02 (1.0–1.03)	0.42	–	–	1.00 (0.98–1.02)	0.71	–	–
BMI	0.99 (0.96–1.02)	0.757	–	–	0.99 (0.95–1.02)	0.61	–	–	1.00 (0.96–1.04)	0.91	–	–
Gender (Female)	**2.18 (1.56–3.04)**	**< 0.001**	**2.20 (1.16–4.2)**	**0.016**	**2.16 (1.48–3.14)**	**< 0.001**	**2.39 (1.20–4.84)**	**0.01**	**2.21 (1.42–3.64)**	**< 0.001**	1.59 (0.67–3.77)	0.28
Vegetable consumption^a^	1.28 (0.65–2.38)	0.528	–	–	2.06 (0.90–5.32)	0.10	–	–	0.71 (0.33–1.57)	0.38	–	–
Brine consumption^a^	1.19 (0.85–1.66)	0.412	–	–	**1.48 (1.00–2.20)**	**0.04**	1.58 (0.82–3.10)	0.16	0.84 (0.53–1.31)	0.44	–	–
Meat consumption^a^	**0.23 (0.14–0.35)**	**< 0.001**	**0.22 (0.14–0.57)**	**< 0.001**	**0.24 (0.15–0.39)**	**< 0.001**	**0.28 (0.13–0.59)**	**< 0.001**	**0.20 (0.12–0.35)**	**< 0.001**	**0.20 (0.07–0.35)**	**< 0.001**
Fat consumption^a^	**7.64 (4.96–12.12)**	**< 0.001**	**6.40 (3.45–12.3)**	**< 0.001**	**8.89 (5.22–16.1)**	**< 0.001**	**6.17 (2.98–13.4)**	**< 0.001**	**6.10 (3.38–11.8)**	**< 0.001**	**6.26 (2.64–15.9)**	**< 0.001**
Tobacco users^b^	**2.17 (1.51–3.14)**	**< 0.001**	**1.64 (0.81–3.35)**	**0.146**	**1.91 (1.26–2.88)**	**< 0.001**	1.44 (0.67–3.13)	0.34	2.70 (1.66–4.32)	< 0.001	2.02 (0.76–5.52)	0.16
Alcohol users^b^	**2.89 (1.90–4.49)**	**< 0.001**	**3.07 (1.48–6.59)**	**< 0.001**	**2.91 (1.82–4.68)**	**< 0.001**	**2.62 (1.16–6.13)**	**0.02**	**2.87 (1.66–4.95)**	**< 0.001**	**3.66 (1.35–10.5)**	**0.01**
Physical activity level^a^	**0.03 (0.02–0.05)**	**< 0.001**	**0.02 (0.01–0.03)**	**< 0.001**	**0.04 (0.02–0.07)**	**< 0.001**	**0.028 (0.01–0.05)**	**< 0.001**	**0.01 (0.00–0.03)**	**< 0.001**	**0.00 (0.00–0.02)**	**< 0.001**
rs3331 (T/T vs. T/C)	**1.78 (1.23–2.58)**	**0.002**	1.82 (0.99–3.38)	0.054	**1.70 (1.12–2.59)**	**0.01**	1.55 (0.79–3.03)	0.19	1.94 (1.16–3.32)	0.01	2.20 (0.92–5.37)	0.07
rs3331 (T/T vs. C/C)	1.15 (0.72–1.85)	0.549	1.56 (0.73–3.37)	0.248	1.03 (0.59–1.78)	0.89	1.38 (0.58–3.28)	0.45	1.38 (0.71–2.65)	0.33	2.16 (0.73–6.60)	0.16
rs1051295 (A/A vs. A/G)	**3.27 (2.14–5.06)**	**< 0.001**	**4.65 (2.28–9.76)**	**< 0.001**	**3.19 (1.97–5.72)**	**< 0.001**	**5.22 (2.38–11.9)**	**< 0.001**	**3.34 (1.89–6.52)**	**< 0.001**	**3.49 (1.34–9.54)**	**0.01**
rs1051295 (A/A vs. G/G)	1.03 (0.64–1.67)	0.875	1.68 (0.79–3.63)	0.176	0.97 (0.56–1.71)	0.93	1.75 (0.75–4.15)	0.19	1.15 (0.58–2.34)	0.68	1.60 (0.56–4.75)	0.38
rs11468831 (ins/ins vs. ins/del)	**0.41 (0.27–0.63)**	**< 0.001**	**0.43 (0.21–0.85)**	**< 0.001**	**0.38 (0.23–0.60)**	**< 0.001**	**0.43 (0.20–0.89)**	**0.02**	**0.49 (0.28–0.85)**	**0.002**	0.42 (0.16–1.05)	0.38
rs11468831 (ins/ins vs. del/del)	**0.34 (0.23–0.51)**	**< 0.001**	**0.27 (0.13–0.51)**	**< 0.001**	**0.30 (0.19–0.47)**	**< 0.001**	**0.24 (0.11–0.50)**	**< 0.001**	**0.44 (0.26–0.74)**	**0.01**	0.65 (0.24–173)	0.06

Value in bold is statistically significant at 5% level of significance

*BMI* Body Mass Index, *OR* Odds Ratio, *CI* Confidence Interval (95%)

*P p* value comparing all controls vs. *C*ases (CRC, RC, CC)

P^a^ *p* value adjusted for CRC and RC *p* value was adjusted for gender, physical activity level, tobacco use, alcohol use, meat and fat consumption, for CC *p* value was adjusted for gender, physical activity level, tobacco use, alcohol use, brine, meat and fat consumption

^a^The reference used was the low consumption level

^b^ The reference used was non-users

### Lifestyle

Significant differences of lifestyle variables (Table 2.) were found between both groups in high meat and fat consumption, tobacco and alcohol use and high physical activity level (*p* < 0.001). Association of lifestyle factors with CRC risk and separately with Colon Cancer (CC) and Rectum Cancer (RC) risk was tested using bivariate and multivariable models. Of co-factors tested using bivariate analysis, a significant association of gender, high meat and high fat consumption, tobacco and alcohol use and high physical activity (p < 0.001) was found with increased CRC risk. However, when tested using a multivariable model, tobacco use was not significantly associated with CRC risk (*p* = 0.146). Female alcohol users with high fat consumption were shown to be at increased risk of CRC (*p* = 0.016). However, for the entire cohort, negative association was found for high meat consumers and high physical activity with CRC risk.

Results of association after stratification by tumor site are detailed in Table 2. Colon Cancer (CC) demonstrated a significant association between gender (*p* < 0.01), high brine (*p* = 0.04), fat consumption, tobacco and alcohol use (*p* < 0.001) with increased risk (OR = 2.16, 1.48, 8.89, 1.91 and 2.91 respectively). However, after multivariate testing, tobacco use and high brine consumption were not significantly associated with CC risk (*p* = 0.16 and *p* = 0.34 respectively). Multivariate testing demonstrated a positive association between females, high fat consumption and alcohol use with CC risk (*p* < 0.01). A negative association is noted between high meat consumption and high level of physical activity with CC risk (*p* < 0.01, OR = 0.24 and 0.04 respectively).

Regarding RC cases, bivariate analysis detects significant association between females, high fat consumption (*p* < 0.001) and alcohol use (*p* = 0.01) with RC risk (OR = 2.21, 6.10 and 2.87). According to the multivariate analysis, there is no significant association between female (*p* = 0.28), tobacco use (*p* = 0.16) and RC risk. A positive association was found between high fat consumption (*p* < 0.001) with a 6-fold increased risk of RC development (OR = 6.26, CI95% [2.64–15.9]), Alcohol users have a 4-fold (3.66) increased risk to develop RC (*p* = 0.01). However, negative association was noted between high meat consumption (OR = 0.20, CI95% [0.07–0.35]), and high physical activity (OR = 0.00, CI95% [0.00–0.02]) and individuals with RC.

### Genetic

Functional prediction of the influence of the three tested polymorphisms (rs3331, rs1051295, and rs11468831) on Kv2.1 are summarized on Table 3. The “highly significant” fitCons scores (*p* < 0.003) indicated likely low fitness of all three variants: rs1051295 (*p* = 0.078); rs11468831 (*p* = 0.065); rs3331 (*p* = 0.053). The GWAVA scores ranged from 0.43 to 0.48 (Region), 0.15 to 0.87 (TSS) and from 0.09 to 0.77 (unmatched) indicating a predicted functional impact of rs3331 and rs1051295 and no predicted functional impact for rs11468831.

**Table 3:** Functional scoring prediction for *KCNB1* SNPs and related Indel

Genotype	FitCons		GWAVA		
	Fitness Score	***P***-value	Region Score	TSS Score	Unmatched Score
**rs3331**	0.053691	< 0.003	0.45	0.87	0.73
**rs1051295**	0.078448	< 0.003	0.43	0.76	0.77
**rs11468831**	0.06567	< 0.003	0.48	0.15	0.09

*SNPs* Single Nucleotide Polymorphisms, *FitCons* Fitness consequence, *GWAVA* Genome Wide Annotation of Variants, *TSS* Transcription Start Site

Allelic distributions of the tested variants among study subjects, as well as stratified analysis according to gender and tumor site are summarized in Table 4. Significant association was found between rs11468831 and CRC occurrence regardless of tumor site and gender (*p* < 0.01). Significant association was also found between major allele of rs3331 “T” and CRC. In fact, carriers of the “T” allele have higher cancer occurrence than “C” allele carriers (*p* = 0.05, OR = 1.08; CI 95% [1.0–1.17]). The association of rs3331 with CRC marginally disappear by cohort stratification according to gender (*p* = 0.18 and *p* = 0.07), as well as according to tumor site (CC and RC), (*p* = 0.08 and *p* = 0.09). No significant allelic association between rs1051295 and CRC risk was detected (*p* = 0.148).

**Table 4:** Allele frequencies of *KCNB1* SNPs and related Indel

SNPs	Total population						Stratification according to sex										Stratification according to tumor site									
	Colorectal cancer (CRC)						Males					Females					Colon Cancer (CC)					Rectum Cancer (RC)				
	Cases ***n*** = 300(%)	Controls ***n*** = 300(%)	P	P^**a**^	OR	CI (95%)	Cases ***n*** = 146(%)	P	P^**a**^	OR	CI (95%)	Cases ***n*** = 154(%)	P	P^**a**^	OR	CI (95%)	Cases ***n*** = 190(%)	P	P^**a**^	OR	CI (95%)	Cases ***n*** = 110(%)	P	P^**a**^	OR	CI (95%)
**rs3331**																										
**T**	325 (54)	344 (57)	0.147	**0.05**	1.00	Ref	154 (53)	0.11	0.18	1.00	Ref	171 (56)	0.325	**0.077**	1.00	Ref	210 (55)	0.283	0.08	1.00	Ref	115 (52)	0.112	0.09	1.00	Ref
**C**^a^	275 (46)	256 (43)			**1.08**	**[1.0–1.17]**	138 (47)			1.10	[0.95–1.27]	137 (44)			1.05	[0.95–1.17]	170 (45)			1.16	[0.82–1.65]	105 (48)			1.09	[0.98–1.22]
**rs1051295**																										
**A**	284 (47)	275 (46)	0.32	0.148	1.00	Ref	146 (50)	0.13	**0.07**	1.00	Ref	138 (45)	0.411	0.18	1.00	Ref	182 (48)	0.29	0.319	1.00	Ref	102 (46)	0.477	**0.07**	1.00	Ref
**G**^a^	316 (46)	325 (54)			0.93	[0.86–1.02]	146 (50)			0.87	[0.75–1.01]	170 (55)			0.93	[0.83–1.03]	198 (52)			0.95	[0.86–1.04]	118 (54)			0.90	[0.81–1.00]
**rs11468831**																										
**ins**	348 (58)	240 (40)	**< 0.01**	**< 0.001**	1.00	Ref	160 (55)	**< 0.01**	**< 0.001**	1.00	Ref	188 (61)	**< 0.01**	**< 0.001**	1.00	Ref	230 (60)	**< 0.01**	**< 0.01**	1.00	Ref	118 (54)	**< 0.01**	**0.005**	1.00	Ref
**del**^a^	252 (42)	360 (60)			**0.80**	**[0.74–0.87]**	132 (45)			**0.747**	**[0.64–0.86]**	120 (39)			**0.83**	**[0.74–0.92]**	150 (40)			**0.79**	**[0.72–0.87]**	102 (46)			**0.85**	**[0.76–0.95]**

Value in bold is statistically significant at 0.05 level of significance

*SNPs* Single Nucleotide Polymorphisms, *Ref* Reference, *OR* Odds Ratio, *CI* Confidence Interval (95%)

*P p* value comparing all Controls vs. *C*ases (CRC, RC, CC)

P^**a**^: *p* value adjusted for CRC and RC *p* value was adjusted for gender, physical activity level, tobacco use, alcohol use, meat and fat consumption, for CC *p* value was adjusted for gender, physical activity level, tobacco use, alcohol use, and for brine, meat and fat consumption

^a^ minor allele

Genotype distributions of the tested variants among study subjects, as well as stratified analysis according to gender and tumor site are shown in Table 5. Of the three polymorphisms analyzed, significant differences in frequency distributions of rs1051295 A/G, rs11468831 ins/del and del/del genotypes (*p* < 0.001) and rs3331 T/C (*p* = 0.005) was found between CRC cases and control subjects, supporting association of these variants to CRC development in Tunisian population confirming the marginal association found in the allelic results.

**Table 5:** Genotype frequencies of *KCNB1* SNPs and related indel

SNPs	Total population					Stratification according to sex								Stratification according to tumor site							
	Colorectal cancer (CRC)					Males					Females			Colon Cancer (CC)				Rectum Cancer (RC)			
	Cases ***n*** = 300(%)	Controls ***n*** = 300(%)	P	OR	CI (95%)	Cases ***n*** = 153(%)	P	OR	CI (95%)	Cases ***n*** = 157(%)	P	OR	CI (95%)	Cases ***n*** = 190(%)	P	OR	CI (95%)	Cases ***n*** = 110(%)	P	OR	CI (95%)
**rs3331**																					
T/T	77 (25.7)	106 (35.3)	**0.005**	**1.00**	**Ref**	**36 (24.7)**	0.47	1.00	Ref	41 (26.6)	**0.012**	**1.00**	**Ref**	**51 (26.8)**	**0.02**	**1.00**	**Ref**	**26 (23.6)**	**0.03**	**1.00**	**Ref**
T/C	171 (57)	132 (44)		**1.78**	**1.23–2.59**	**82 (56.1)**		1.44	0.78–2.68	89 (57.8)		**1.92**	**1.19–3.12**	**108 (56.8)**		**1.70**	**1.12–2.59**	**63 (57.3)**		**1.94**	**1.16–3.23**
C/C^a^	52 (17.3)	62 (20.7)		1.15	0.72–1.84	28 (19.2)		1.14	0.53–2.47	24 (15.6)		1.06	0.56–1.98	31 (16.4)		1.03	0.59–1.78	21 (19.1)		138	0.71–2.65
**rs1051295**																					
A/A	46 (15.3)	84 (28.0)	**< 0.001**	**1.00**	**Ref**	**25 (17.1)**	**< 0.001**	**1.00**	**Ref**	**21 (13.6)**	**< 0.001**	**1.00**	**Ref**	**30 (15.8)**	**< 0.001**	**1.00**	**Ref**	**16 (14.6)**	**< 0.001**	**1.00**	**Ref**
A/G	192 (64)	107 (35.7)		**3.27**	**2.14–5.06**	**96 (65.8)**		**3.71**	**1,90–7.35**	**96 (62.4)**		**3.26**	**1.84–5.96**	**122 (64.2)**		**3.19**	**1.97–5.27**	**70 (63.6)**		**3.43**	**1.89–6.52**
G/G^a^	62 (20.7)	109 (36.3)		1.03	0.64–1.67	25 (17.1)		0.90	0.42–1.91	37 (24.0)		1.25	0.66–2.40	38 (20)		0.97	0.56–1.71	24 (21.8)		1.15	0.58–2.34
**rs11468831**																					
ins/ins	135 (45)	71 (23.7)	**< 0.001**	**1.00**	**Ref**	**59 (40.4)**	**0.01**	**1.00**	**Ref**	**76 (49.4)**	**< 0.001**	**1.00**	**Ref**	**91 (47.9)**	**< 0.001**	**1.00**	**Ref**	**44 (40)**	**0.004**	**1.00**	**Ref**
ins/del	78 (26)	98 (32.7)		**0.41**	**0.27–0.63**	**42 (28.2)**		0.61	0.30–1.2	36 (23.4)		**0.32**	**0.18–0.55**	**48 (25.3)**		**0.38**	**0.23–0,60**	**30 (27.3)**		**0.49**	**0.28–0.85**
del/del^a^	87 (29)	131 (43)		**0.34**	**0.23–0.51**	**45 (30.8)**		**0.39**	**020–0.75**	**42 (27.2)**		**0.30**	**018–0.50**	**51 (26.8)**		**0.30**	**0.19–0.47**	**36 (32.7)**		–	0,44 (2–0,8)

Value in bold is statistically significant at 0.05 level of significance

*SNPs* Single Nucleotide Polymorphisms, *Ref* Reference, *OR* Odds Ratio, *CI* Confidence Interval (95%),

*P p* value comparing healthy controls vs. *C*ases (CRC, RC, CC)

^a^ minor genotype

No significant differences were found when cases were stratified by tumor site (CC and RC). In fact, heterozygous genotype of both SNPs: rs3331 T/C and rs1051295 A/G were positively correlated to CRC development regardless of malignancy localization (*p* = 0.02 and *p* = 0.03). As such, carriages of the rs3331 unfavorable genotype are with 2-folds (1.92 and 1.70 respectively) with CRC development. Carriers of one copy of rs1051295 (A/G) are 3-fold higher susceptible to develop CRC. Furthermore, the homozygous major genotype of rs11468831 ins/ins is associated with CRC occurrence regardless of tumor site (RC and CC) and patients’ carriers of non-ins/ins genotype are less likely to install colorectal cancer (*p* < 0.01) (Table 5).

The KCNB1 rs3331 and rs1051295 haplotype shows no association to CRC, CC and RC susceptibility in the Tunisia population (data not shown).

### Genetic and lifestyle

The correlation between *KCNB1* variants (rs3331, rs1051295 and rs11468831) and five subjects’ lifestyle factors (meat and fat consumption, tobacco and alcohol use and physical activity level) were analyzed (Table 2) for CRC and the segregated RC and CC cohorts. In addition, analyses were conducted after adjustment to those five factors for CRC and RC and for those five factors plus brine consumption for CC. Carriage of rs1051295 A/G was still associated with increased risk of CRC, CC (*p* < 0.001) and RC (*p* = 0.01). The rs11468831 ins/del genotype was negatively associated with risk for CRC (*p* < 0.001) and CC (*p* = 0.02) and not associated with RC risk (*p* = 0.38). The rs11468831 del/del genotype was negatively associate with risk of CRC, and CC (*p* < 0.001) and not associated with RC (*p* = 0.06). On the other hand, the rs3331 T/C genotype was not associated to CRC, CC nor RC.

The risk of CRC, CC and RC associated with *KCNB1* genotypes and the related indel after adjustment for co-factors such as gender, physical activity, tobacco and alcohol use, brine, meat and fat consumption was assessed using the multivariable logistic regression model. The occurrence of CRC and CC was found to be associated with rs11468831 under the codominant (*p* < 0.001) and dominant (*p* < 0.001 and *p* = 0.02) respectively. In addition, rs1051295 was found to be associated under the codominant (*p* < 0.001) only. The association of rs1051295 with both CRC and CC was seen only in heterozygous carriers A/G, and for rs11468831 was observed in heterozygous ins/del and homozygosis del/del. In RC cases, rs1051295 was found to be highly associated under the codominant (*p* < 0.001) only. The association of rs1051295 with RC was seen only in heterozygous carriers A/G.

## Discussion

The etiology of CRC remains elusive and most likely includes lifestyle, physiology, and genetic factors and their interaction. In Tunisia, fundamental risk factors of CRC such as clinical history, genetics and lifestyle are still not well understood and therefore the contribution of important but diffuse factors such as socioeconomic status (SES) and environmental risk remain unknown. In addition, Tunisia has recently experienced profound Western culture related changes in lifestyle and an increase in new colon and rectal cancer cases. Indeed, the heterogeneity of this malignancy and the variability observed in different populations suggests the need for a Tunisian study of select individual and family clinical history, lifestyle and genetic factors. This is the first Tunisian study to test the effect of a wide collection of factors representative of Western lifestyle including diet, tobacco and alcohol use, and physical activity habits on CRC susceptibility and the first to characterize and test association with variants of an important cancer-implicated voltage-gated potassium channel Kv2.1.

Our results report about twice high frequency of CC compared to RC (66.3 vs 36.7%) in the Tunisian population a result consistent with earlier studies [26]. Part of the cancer site variation may be attributed to the recent change in the Tunisian diet from a largely Mediterranean to a more heavily influenced Western diet. Several studies have consistently confirmed a correlation between high meat consumption, even in low or middle-income Westernized countries and CRC susceptibility [27]. Interestingly, the percent of CRC cases who were high versus low meat consumers in Tunisia was significantly low (42.3% vs. 57.6%, *p* < 0.001) and that 89% of controls were high meat consumers (Table 1). In Tunisia, a Mediterranean middle-income country, the high consumption of meat is characterized by significant amounts of fish and poultry [28]. Fish consumption is negatively correlated with CRC risk [27] suggesting why our study demonstrates that high meat consumption is associated with lower CRC risk contrary to meat consumption association with increased CRC risk established in studies of other populations [29, 30]. Further studies are required to fully elucidate these findings.

The Development of CRC undergoes four stages: initiation (stage I), promotion (stage II), progression (stage III) and metastasis (stage IV). In our study, 81% of cases are Stage II and III, while 18.7% are Stage IV. In addition, 90.3% of all cases classified as high fat consumers whereas 55% of healthy controls were high fat consumers. Combined, these results are consistent with known high fat diet’s effect on eliciting variation of intestinal stem cells and influence on increased tumorigenicity of intestinal progenitors [31, 32]. Our analysis demonstrates comparable BMI (*p* = 0.75) between cases and controls (25.04 and 25.6, *p* = 0.75, Table 1) and a reduction of CRC risk with high physical activity. Suggesting that a high fat diet and obesity may be mitigated by high physical activity.

The sex-ratio (SR) 0.94 of cases was opposite to other studies demonstrating male predominance in CRC incidences [33–35]. This contrast may be explained by recent Tunisian female work and lifestyle changes that suggest occupational and environmental risk factor exposure comparable with Tunisian males.

Our Tunisian study also establishes that high alcohol use is associated with CRC occurrence regardless of neoplasia site a result consistent with results in high-income country studies [26]. Such consistency of risk may be attributed to alcohol’s first metabolite (acetaldehyde) classification as a human carcinogenic by the IAR [36]. Ingested alcohol is metabolized into acetaldehyde resulting in mucosal injuries and regenerative cellular proliferation [37, 38] and accumulation in the intestinal epithelial cells therein possibly promoting carcinogenesis through DNA damage [37, 38]. We find no association between tobacco use and CRC development and a larger percentage of CRC case tobacco non-users compared to users (63.3% vs. 36.7%, *p* < 0.001).

Alterations in tumoral microenvironment affected by lifestyle changes play an important role in tumor progression via cell-cell interactions, cell-matrix signals and other related voltage gated potassium channels (Kv) related molecular function [39]. The Kv2.1 channel has been shown to play a role in these physiological processes and therefore is a compelling channel to study in site-specific CRC, CC and RC occurrence and/or metastasis. In addition, Kv2.1’s heterotetramerization with other channels subunits increases channel diversity and therefore the potential deleterious role during the malignancy process [40]. For example, the Kv9.3 channel (an electrophysiological silent subunit) expression increased in CRC and is a heterotetramer with Kv2.1 suggesting, the combination may modify electrophysiological properties in CRC [14]. These findings suggest that Kv9.3 (without Kv2.1) plays a role in cell-cycle progression and cell proliferation. However, the observed effects on cell-cycle progression and cell proliferation may also be caused by a changed ratio of free Kv2.1/Kv9.3 hetero- and Kv2.1 homotetramers secondary to the reduced Kv9.3 expression [14]. Other evidence suggests a mechanistic basis for the modulation of Kv2.1 channel inactivation gating kinetics by silencing the Kv6.4 subunits, associated with CRC [14], via the co-assembles of Kv2.1/Kv6.4 [41].

Interestingly, it has been suggested that the Kv2.1 channel may function as a promoting signal for focal adhesion kinase (FAK) activation which is associated with CRC metastasis [42, 43]. In fact, the Kv2.1 channel provides a unique voltage-dependant mechanism underlying FAK activation in response to microenvironment changes and a wide variety of pathophysiological conditions such as formation of tumor metastasis and FAK motility [44]. Together, clustering Kv2.1/FAK promotes increased invasiveness of CRC cells. Furthermore, as colon cancer cells are known to usurp excitable cells to facilitate invasion and due to Kv2.1 ability in modulating the electrophysiology of excitable cells it is plausible that Kv2.1 could be related to tumor progression. The surprising variability of Kv2.1 function within the microenvironment highlights the possible if not likely importance of such channels in CRC and suggests additional studies to discover Kv2.1 function that may be modified adversely with Western influenced lifestyles such as increased fat and meat consumption. In addition, our recent results showed that *KCNB1* polymorphisms were associated with Tumor-Node-Metastasis (TNM) progression and treatment response and suggests that such polymorphisms as biomarkers of CRC outcomes, before and during chemotherapy process [17]. The accumulated body of evidence of the importance and functional contribution of Kv2.1 in cancer suggested the importance of testing variants’ of *KCNB1* (the Kv2.1 coding gene) association with CRC, CC and RC. Indeed, our study demonstrates important association between site-specific digestive cancers and functionally impactful genetic variation found in the *KCNB1* gene. In particular, the A allele of rs1051295 was predicted to be located in the has-miR-216a binding site suggesting the presence of the G allele may increase *KCNB1* expression, thereby inhibiting tumor progression [44] a result consistent with the presence of more G alleles in our study’s Controls versus Cases. We also found that the rs11468831 indel variant is protective against CRC risk regardless of tumor site. The association of functionally impactful *KCNB1* variants and CRC susceptibility in the Tunisian population are consistent with the *proven involvement of the KCNB1 gene in the autophagy process that inhibits tumor growth and proliferation* [45]*. The* established role of*, KCNB1* modulation on the autophagy mechanism and may be important in CRC’s aggressiveness and metastasis [46–50].

## Conclusion

Our results demonstrate significant association between diet, alcohol and tobacco use, *KCNB1* gene variations and CRC development in the Tunisian population. Though non-targeted CRC screening programs are expensive for population-wide use in Tunisia, public health concerns and CRC predictions based on changing lifestyles are important to government policies seeking CRC incidence reduction. Such policies benefit from this and related studies of the role of modifiable risk factors that provide evidence and context informing less costly targeted screening based on known Tunisian Western-influenced lifestyle changes. Future efforts will include studies of environment perturbations, additional ion channel examination and Tunisian specific lifestyle designed to guide and provide insight into more precise screening, new therapeutics and improved CRC management.

## Data Availability

The data sets used in this study are available from the corresponding author.

## Abbreviations

3’UTR: Three prime UnTranslated Region
ARM-PCR: Amplification-refractory mutation system-Polymerase chain reaction
BMI: Body Mass Index
*BRAF*: Serine/ theonine-protein kinase B-Raf
CA19–9: Carbohydrate antigen 19–9
CAE: Carcinoembryonic antigen
DNA: DeoxyriboNucleic Acid
DCP DB: Digestive Cancer Project Data Base
EPIC: European Prospective Investigation into Cancer and Nutrition
ESMO: European Society for Medical Oncology
IARC: The International Agency for Research on Cancer.
fitCons: Fitness consequence.
GWAVA: Genome Wide Annotation of VAriants.
GWAS: Genome Wide Association Study.
*KCNB1*: Potassium Voltage-gated channel subfamily B member 1.
Kv2.1: Voltage-gated potassium channel, Shab-related subfamily, member 1.
Kv9.3: Voltage-gated potassium channel subunit Kv9.3
Kv6.2: Voltage-gated channel potassium subunit Kv6.2
MAF: Minor Allele Frequency
OR: Odds Ratio
PCR: Polymerase chain reaction
ReMM: Regulatory Mendelian Mutation
SNPs: Single Nucleotide Polymorphisms
TNM: TNM Classification of Malignant Tumors
TSS: Transcription Start Site
*TP53*: Tumor protein p53
USPSTF: United States Preventive Services Task Force
WHO: World Health Organization
